# The perceptions and experiences of community nurses and patients towards shared decision‐making in the home setting: An integrative review

**DOI:** 10.1111/jan.16345

**Published:** 2024-07-22

**Authors:** Katie Mills, Lucy McGeagh, Marion Waite, Helen Aveyard

**Affiliations:** ^1^ Oxford School of Nursing and Midwifery, Faculty of Health and Life Sciences Oxford Brookes University Oxford UK

**Keywords:** community nursing, informed consent, integrative review, nurse–patient interaction, nurse–patient relationship, patient experience, patient participation in decision‐making, person‐centred care, shared decision‐making

## Abstract

**Aim:**

To explore patients' and community nurses' perceptions and experiences of shared decision‐making in the home.

**Design:**

Integrative review.

**Data Sources:**

CINAHL, British Nursing Index, Psycinfo, Medline and Social Services Abstracts were searched for qualitative, quantitative and mixed methods papers published between 1 December 2001 and 31 October 2023.

**Review Methods:**

A systematic search of electronic databases was undertaken using defined inclusion criteria. The included papers were appraised for quality using the Joanna Briggs Institute critical appraisal checklist for qualitative research. Relevant data were extracted and thematically analysed.

**Results:**

Fourteen papers comprising 13 research studies were included. Patients attached great importance to their right to be involved in decision‐making and noted feeling valued as a unique individual. Communication and trust between the patient and nurse were perceived as fundamental. However, shared decision‐making does not always occur in practice. Nurses described tension in managing patients' involvement in decision‐making.

**Conclusion:**

The findings demonstrate that although patients and community nurses appreciate participating in shared decision‐making within the home, there are obstacles to achieving a collaborative process. This is especially relevant when there are fundamentally different perspectives on the decision being made. More research is needed to gain further understanding of how shared decision‐making plays out in practice and to understand the tensions that patients and nurses may experience.

**Implications for the Profession and/or Patient Care:**

This paper argues that shared decision‐making is more than the development of a relationship where the patient can express their views (though of course, this is important). Shared decision‐making requires acknowledgement that the patient has the right to full information and should be empowered to choose between options. Nurses should not assume that shared decision‐making in community nursing is easy to facilitate and should recognize the tensions that might exist when true patient choice is enabled.

**Impact:**

This paper demonstrates how the idea of shared decision‐making needs to be explored in the light of everyday practice so that challenges and barriers can be overcome. In particular, the tensions that arise when patients and nurses do not share the same perspective. This paper speaks to the potential of a gap surrounding shared decision‐making in theory and how it plays out in practice.

**Reporting Method:**

The reporting of this review was guided by the 2020 guidelines for the Preferred Reporting Items for Systematic Reviews and Meta‐Analyses (Page et al., 2021).

**Patient or Public Contribution:**

This review was carried out as part of a wider study for which service users have been consulted.

**What does this paper contribute to the wider global clinical community?**

- There is insufficient evidence that shared decision‐making in community nursing in the home is a global reality.
- There is evidence of tension that exists when patients' and nurses' views towards a decision are different.
- A gap is evident in the literature as to how the process of shared decision‐making between the community nurse and patient plays out in the home.

## 1 INTRODUCTION

Shared decision‐making between the patient and healthcare professional involves the mutual sharing of information and a decision made in partnership based on the evidence base and the patient preferences and values (Elwyn et al., 2017). Recognition of the importance of involving patients in the decision‐making process is well established within nursing practice. However, it remains unclear how shared decision‐making happens in practice (Marriott‐Statham et al., 2023), particularly within the context of nursing care within the patient's home. In this review, we explore the existing literature, focusing on the experiences and perceptions of patients and nurses towards shared decision‐making in the home.

## 2 BACKGROUND

### 2.1 Shared decision‐making and informed consent

The decision‐making interaction between the patient and healthcare professional is grounded by the principles of informed consent, which are reflected in the ethical process of shared decision‐making. The principles of informed consent are well established in both the law and ethics in many countries and include thorough disclosure of information about the treatment, understanding of the information, voluntariness to make the decision and mental capacity (Bolcato et al., 2024; Faden et al., 1986). Shared decision‐making is underpinned by and evolved from informed consent. The concept stemmed from the President's Commission for the Study of Ethical Problems in Medicine and Biomedical and Behavioural Research (1982) in the United States where the recommendation was made that ‘ethically valid consent is a process of shared decision‐making based upon mutual respect and participation’ (p. 2).

In the United Kingdom, the case of ‘Montgomery v. Lanarkshire Health Board (2015)’ established the centrality of the patient's values and preferences within the consent process (Chan et al., 2017; Ward et al., 2020). While there is debate in the literature around the implications of the Montgomery ruling (Le Gallez et al., 2022), there is recognition that, in the United Kingdom, the Montgomery ruling has moved shared decision‐making from professional guidance to a legal requirement (Ward et al., 2020).

### 2.2 Benefits of shared decision‐making on patient outcomes

Shared decision‐making has been found to have a beneficial impact on patient outcomes including improving people's healthcare knowledge and self‐confidence, involvement in and satisfaction with their care and increasing healthcare professional's communication skills (Health Foundation, 2012; National Institute for Clinical Excellence (NICE), 2021; Shay & Lafata, 2015). The benefits of shared decision‐making have been emphasized for people from disadvantaged backgrounds (Coulter & Collins, 2011) including increased knowledge, informed choice and increased decision self‐efficacy (Durand et al., 2014). A survey by The Patients Association (2021) found that service users valued the process of involvement in decision‐making including the need for trust, being listened to and understanding the problem that they were deliberating, making a decision and recognizing language and cultural barriers. Within the district nursing service in the United Kingdom, Maybin et al. (2015) found that service users, carers and community nurses shared values about the importance of involving people in decisions about their care.

There is a clear aspiration to involve patients in the decision‐making process, as demonstrated within health policy. In the United Kingdom, for at least two decades, the importance of shared decision‐making has been emphasized, most recently in the NHS Long‐Term Plan (NHS England, 2019). The Nursing and Midwifery Council acknowledges the professional responsibility of nurses in the United Kingdom to facilitate patients' engagement in decision‐making. The Professional Code of Conduct for registered nurses (RNs) states that RNs must ‘encourage and empower people to share in decisions about their treatment and care’ (Nursing Midwifery Council, 2018, p. 7).

### 2.3 Shared decision‐making models

Shared decision‐making depends on building a trusting relationship between the patient and the healthcare professional. Thus enabling decisions to be made by exploring and respecting what matters most to patients as individuals (Elwyn et al., 2012). McCormack and McCance (2017) included shared decision‐making as one of the nursing processes within their person‐centred practice framework. They highlighted the importance of the therapeutic relationship within the shared decision‐making process to enable negotiation to take place recognizing the patients’ beliefs and values. Makoul and Clayman (2006) developed a framework of shared decision‐making including nine elements: defining the problem, presenting options, discussing pros and cons, patient values and preferences, discussing patient ability or self‐efficacy, health professionals’ recommendations, make or defer decisions and arranging follow‐up. Elwyn et al.'s (2017) circular three‐talk model of shared decision‐making, emphasizes the communication that occurs between the patient and healthcare professional. This model includes three stages of ‘*team talk*’, working together and discussing goals, ‘*option talk*’, discussing alternative treatments and ‘*decision talk*’, reaching a preference‐based decision. Active listening and deliberation are at the centre of the model.

### 2.4 Patients and nurses' experiences of shared decision‐making

Despite the emphasis on shared decision‐making within the legal, theoretical and policy literature, the extent to which patients are involved with decisions in practice is less clear and the concept of shared decision‐making is not always well understood by patients and healthcare professionals. The Patients Association (2021) report on shared decision‐making found that service users did not always understand the term describing it as ‘*jargon*’ (p. 9) although it was clear that they valued involvement in decision‐making.

Previous systematic literature reviews have found that patients and healthcare professionals perceive communication and relationships as necessary to facilitate shared decision‐making (Joseph‐Williams et al., 2014; Kuosmanen et al., 2021; Truglio‐Londrigan & Slyer, 2018; Waddell et al., 2021). However, Pollard et al. (2015) found a lack of consistency between healthcare professionals' expressed positive attitudes towards shared decision‐making, and how decision‐making plays out in practice. Barriers included: time and logistical issues, perceptions of roles and the traditional hierarchy between patients and healthcare professionals.

Previous reviews examining the perceptions and experiences of healthcare professionals and patients of shared decision‐making have predominantly conveyed perspectives on the decision‐making process between doctors and patients (Clark et al., 2009; Légaré et al., 2008; Marriott‐Statham et al., 2023; Pollard et al., 2015; Truglio‐Londrigan & Slyer, 2018). The perspective of nurses, and of patients making decisions with nurses, is limited as well as a lack of knowledge about how shared decision‐making is carried out between nurses and patients (Marriott‐Statham et al., 2023). Through their concept analysis of shared decision‐making theoretical models, Lewis et al. (2016) emphasized the need for more articulation of the nursing role within the shared decision‐making process. There is a clear gap within the literature concerning the perceptions and experiences of nurses and patients in relation to shared decision‐making.

### 2.5 Decision‐making in the home setting

In this review, we focused on shared decision‐making between community nurses and patients in the home. We define the ‘home’ as the residence in which the person lives excluding institutional settings such as a hospice or nursing home. Home nursing, for example, the district nursing service in the United Kingdom, has an important role in supporting people with complex conditions (QNI and RCN, 2019). There are wide variations worldwide as to the availability of home nursing services. Yu et al. (2024) found that there was a higher utilization of home nursing services by older people within Europe than in Asia or the United States. Home nursing services have evolved over the last 10 years, particularly during and after the COVID‐19 pandemic, with an increase in telemedicine and a focus on supporting self‐care. However, there is still a substantial need for in‐person home nursing. For example, in January 2024, in England, there were over two million patient contacts made with the district nursing service, of which 84% were aged 65 years and above (NHS England, 2024).

The nature of nursing in the home will influence the decision‐making process between the nurse and the patient. The types of decisions made within the home might include treatment decisions, management of symptoms, lifestyle choices and referrals to other professionals. The person's home is their personal and private space and the nurse enters the space as a guest. While it could be surmised that making decisions within their private and personal space enables patients to feel more autonomous and to contribute to engagement in decision‐making, there is limited knowledge to support this assumption. Carolan et al. (2005) suggested that perceptions of increased autonomy may alter as the home becomes increasingly medicalized. The patient may choose to involve their family in decision‐making yet if their preference is to make decisions independently there could be challenges in relation to privacy in the home. The home may not always have positive associations for the person and could be risky, for example, where domestic violence is present (Driessen et al., 2021). We could find no previous reviews that focused on the perceptions and experiences of nurses and patients of decision‐making in the home. Several similar reviews have been carried out including all clinical settings with limited discussion on the perspective of the home (Clark et al., 2009; Légaré et al., 2008; Pollard et al., 2015; Truglio‐Londrigan & Slyer, 2018). Reviews have also been carried out focused on the specific clinical areas of palliative care (Kuosmanen et al., 2021) and acute care (Waddell et al., 2021). In this review, we aim to reveal current knowledge about the perceptions and experiences of patients and nurses towards the decision‐making process in the home. Developing an understanding of nurses' and patients' perspectives and experiences is important given the benefits of shared decision‐making together with nurses' legal, professional and ethical requirements to facilitate shared decision‐making. This insight could help to develop strategies supporting nurses to facilitate patients' involvement in decision‐making and to enhance patient outcomes.

## 3 INTEGRATIVE LITERATURE REVIEW

### 3.1 Aims

In this integrative literature review, we aim to explore the perception and experiences of community nurses and patients towards shared decision‐making in the home.

### 3.2 Design

We used Whittemore and Knaffl's (2005) methodology, including the five stages of problem identification, literature search, data evaluation, data analysis and presentation. Whittemore and Knaffl (2005) developed the integrative review methodology to address the complexity of a mixed methods review, which they argued had previously not been well defined. Furthermore, this methodology promotes rigour and reduces bias about analysis, synthesis and conclusion drawing. We were guided in the reporting of this review by the 2020 guidelines for the preferred reporting items for systematic reviews and meta‐analyses (PRISMA) (Page et al., 2021).

### 3.3 Methods

#### 3.3.1 Literature search

We performed a systematic search of primary research studies, qualitative, quantitative or mixed methods, published in the English language between 1 January 2001 and 31 October 2023. No limits were placed on the country of origin. We chose the year 2001 as the start date as the National Service Framework (NSF) for Older People (Department of Health, 2001) was published in the United Kingdom in 2001. The NSF for Older People (Department of Health, 2001) highlighted the importance of supporting older people to make decisions about their own care, encouraging a shift towards involving patients in decision‐making. To construct the question for the review and identify the key terms for the literature search, we used the qualitative mnemonic SPIDER which relates to: sample, phenomenon of interest, design, evaluation and research type, (Cooke et al., 2012). Table 1 shows how SPIDER was used to develop the search terms. Key search terms were ‘decision‐making’, ‘nurse–patient interaction’ and ‘community nursing’. We used synonyms, including recognizing international words used for community nursing, for example, home health nursing and truncation. We combined the search terms using the Boolean operators AND/OR, see Table 2. We searched the databases CINAHL, British Nursing Index, Psycinfo, Medline and Social Services Abstract.

**TABLE 1:** SPIDER and search terms.

	SPIDER terms	Search terms
Sample	Adults receiving nursing care at home Community nurses providing the care at home	Community nurs* District nurs* Home health nurs*
Phenomenon of interest	Nurse–patient interaction in the decision‐making process	Nurse–patient interaction Nurse–patient communication Patient involvement Patient participation Patient engagement Decision‐making Decision‐making Decision‐making process Decision‐making process Shared decision making Shared decision‐making SDM
Design	Observational research. Focus groups or interviews. Qualitative or quantitative survey research design	
Evaluation	Description of nurse–patient interaction Experiences and perceptions of nurse and patient	
Research type	Qualitative research design, for example, ethnography or phenomenology Possible quantitative research design, for example, survey type research Possible mixed methods research design	

**TABLE 2:** Search terms combined with Boolean operators.

Decision making OR Decision‐ making OR Decision making process OR Decision‐making process	AND	Nurse–patient interaction OR Nurse–patient communication OR Patient involvement OR Patient participation OR Patient engagement OR Shared decision making OR Shared decision‐making OR SDM
AND		
Community nurs* OR District nurs* OR Home health nurs*		

We included papers that focused on the perceptions and experiences of adult patients and/ or community nurses of shared decision‐making in the home setting. Studies related to shared decision‐making in either the question or as part of the findings of the study. In some studies, the authors did not mention the specific terms of shared decision‐making or participation in decision‐making, but described these terms in different words with a similar meaning, for example, the phrase used by Olaison et al. (2021): ‘opportunity to participate in and influence how care and treatment should be performed’ (p. 8). We decided to keep a broad focus on decision‐making rather than narrowing it down to a specific type of decision. This was to ensure that we captured all the relevant research papers that focused on shared decision‐making between nurses and patients in the home. We included papers where the data collected related to the home. Institutional settings such as a clinic or nursing home were excluded from the study. Where this was not clear the authors of the papers were contacted to check that the data related to the home. Table 3 sets out the inclusion/ exclusion criteria. We hand‐searched reference lists of included papers which yielded two additional papers.

**TABLE 3:** Inclusion and exclusion criteria.

Inclusion criteria	Exclusion criteria
Studies from 2001	Studies prior to 2001
Primary research studies either qualitative or quantitative or mixed methods	Theoretical papers, policy documents or opinion articles or papers that are not empirical research
Worldwide studies	
English language	Non‐English language
The person is receiving nursing care in the home setting	Receiving nursing care in all settings other than the home, e.g. hospital, clinic
Study focuses on the perceptions and experiences of the community nurse and/ or patient in the home setting	Studies not focused on perceptions and experiences of the community nurse and/ or the patient in the home setting
The study relates to patient participation in decision‐making process or shared decision‐making in the question or the findings Where a study does not mention the specific terms above but has described these terms using different words with a similar meaning the papers were included	The study is not related to patient participation in the decision‐making process or shared decision‐making process in the question or the findings
Adult patients 18 years and over who had the mental capacity to give informed consent	Children or teenagers under 18 years or adults who did not have the mental capacity to give informed consent
Nurses working within the district nursing teams or specialist community nursing teams or equivalent role in a worldwide paper, e.g. home health nurses	Children, health visiting or community learning disability or mental health teams

#### 3.3.2 Search outcome

**FIGURE 1:**
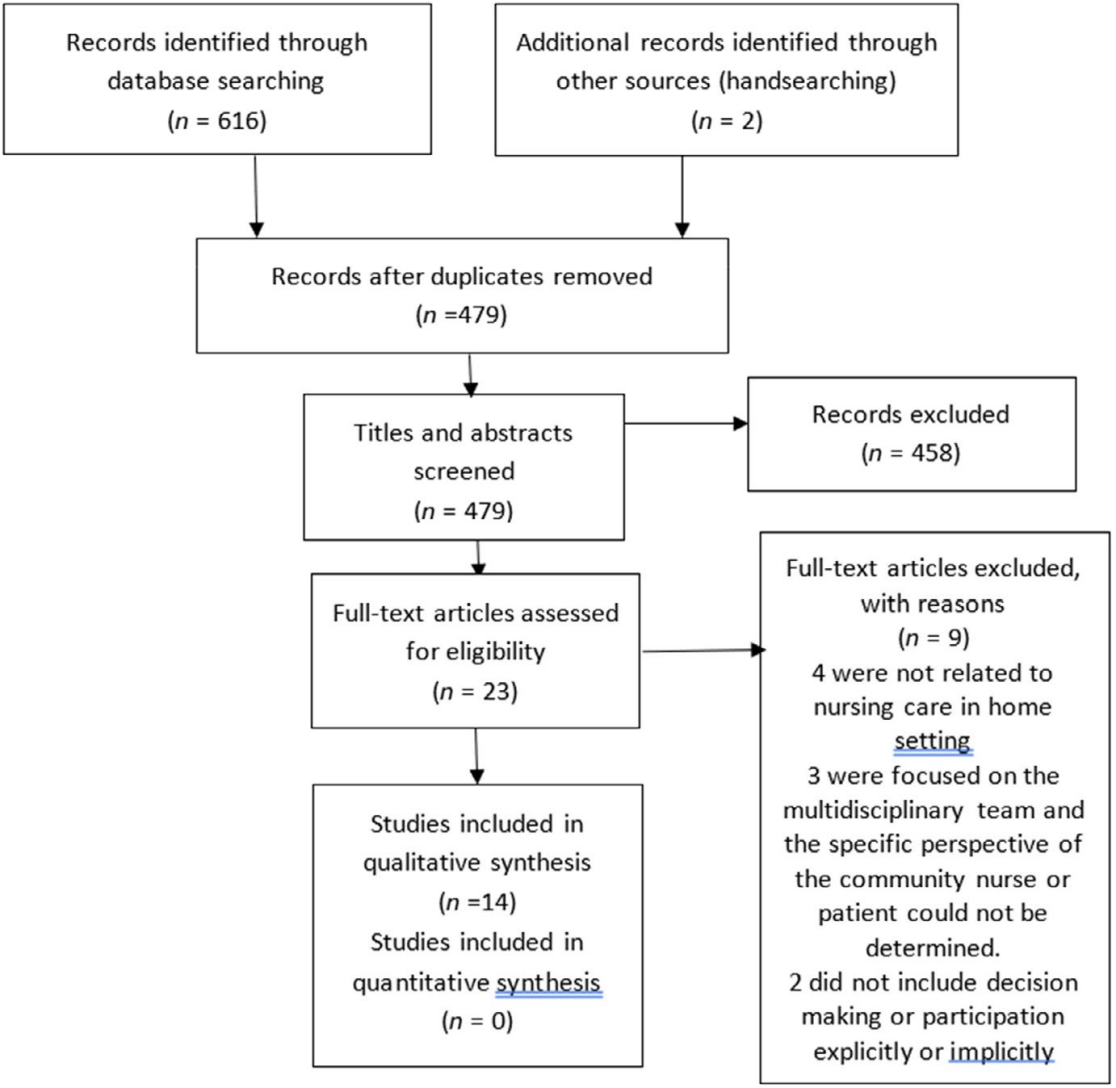
Preferred Reporting Items for Systematic Reviews and Meta‐Analyses flow diagram of screening and exclusion process. Adapted from Moher et al. (2009).

The search produced 479 results after duplicates were removed. As lead author, (KM) screened the papers initially using the title and abstract, excluding 458 papers. All four authors (KM, LM, MW and HA) then independently screened the remaining 23 full‐text papers against the inclusion/ exclusion criteria and following discussion, a consensus was reached to include 14 papers. Two of the papers related to the same study so there were 13 research studies overall. The PRISMA diagram is shown in Figure 1.

#### 3.3.3 Quality appraisal

All of the papers included in this review had a qualitative research design. We used the Johanna Briggs Institute (JBI) critical appraisal checklist for qualitative research (Lockwood et al., 2015) to evaluate their quality. This tool comprises 10 items aimed at appraising the methodological quality of a study and whether the study has addressed the possibility of bias (Lockwood et al., 2015). We judged each item: yes, no, unclear or partially. We created a scale to estimate the quality of the study. If the study received eight or more ‘yes’ responses, it was judged as ‘good’ quality. If it received between 6 and 7 ‘yes’ responses, it was judged as ‘medium’ quality and 5 or lower ‘yes’ responses it was seen as ‘poor’ quality. We made the decision that good or medium‐quality papers would be included within the review and poor‐quality papers would be excluded.

One author (KM) reviewed the methodological quality of each of the papers initially. Then a third of the papers were independently reviewed by a further author (HA). We assessed 13 of the papers as good quality, and one paper as medium quality. No papers were excluded from the study based on quality. The quality assessment results are shown in Table 4.

**TABLE 4:** Quality assessment of the 14 papers using the Johanna Briggs Institute Critical Appraisal checklist.

	JBI checklist question	Baik et al. (2020)	Brogan et al. (2018)	Dickson et al., 2017	Holmberg et al. (2012)	McGarry (2008)	McGarry (2010)	Millard et al. (2006)	Näsström et al. (2013)	Nilsen et al. (2021)	Olaison et al. (2021)	Oliver et al. (2018)	Sundler et al., 2020	Truglio‐Londrigan (2013)	Van Het Bolscher‐Niehuis et al. (2020)
1	Is there congruity between the stated philosophical perspective and the research methodology?	Yes	Yes	Yes	Yes	Yes	Yes	Unclear	Yes	Yes	Unclear	Yes	Unclear	Unclear	Yes
2	Is there congruity between the research methodology and the research question or objectives?	Yes	Yes	Yes	Yes	Yes	Yes	Yes	Yes	Yes	Yes	Yes	Yes	Yes	Yes
3	Is there congruity between the research methodology and the methods used to collect data?	Yes	Yes	Yes	Yes	Yes	Yes	Yes	Yes	Yes	Yes	Yes	Yes	Yes	Yes
4	Is there congruity between the research methodology and the representation and analysis of data?	Yes	Yes	Yes	Yes	Yes	Yes	Yes	Yes	Yes	Yes	Yes	Yes	Yes	Yes
5	Is there congruity between the research methodology and the interpretation of results?	Yes	Yes	Yes	Yes	Yes	Yes	Yes	Yes	Yes	Yes	Yes	Yes	Yes	Yes
6	Is there a statement locating the researcher culturally or theoretically?	No	No	No	Yes	Yes	Yes	No	Yes	Yes	No	No	No	Yes	No
7	Is the influence of the researcher on the research, and vice‐versa, addressed?	No	No	No	No	Yes	Yes	No	Yes	Yes	Yes	No	Yes	Yes	No
8	Are participants, and their voices, adequately represented?	Yes	Yes	Yes	Yes	Yes	Yes	Yes	Yes	Yes	Yes	Yes	Yes	Yes	Yes
9	Is the research ethical according to current criteria or, for recent studies, and is there evidence of ethical approval by an appropriate body?	Yes	Yes	Yes	Yes	Yes	Yes	Yes	Yes	Yes	Yes	Yes	Yes	Yes	Yes
10	Do the conclusions drawn in the research report flow from the analysis, or interpretation, of the data?	Yes	Yes	Yes	Yes	Yes	Yes	Yes	Yes	Yes	Yes	Yes	Yes	Yes	Yes
Quality assessment results		Good	Good	Good	Good	Good	Good	Medium	Good	Good	Good	Good	Good	Good	Good

#### 3.3.4 Data extraction

One author (KM) extracted data from the 14 papers relating to 13 research studies that met the inclusion criteria. Only data relevant to the review question were included. Then a third of the papers were independently reviewed by a further author (HA) to check the data extraction accuracy. Table 5 shows the data extraction of the research studies, including the main study characteristics and findings.

**TABLE 5:** Data extraction table (based on the format of Stayt & Nemes, 2018).

Author	Research aim	Sample	Design	Outcome measures/data generation	Data analysis	Results/findings (that relate to the review question)	Summary of study evaluation (see Table 4)
Baik et al. (2020)	To investigate the barriers and facilitators that shape goals of care conversations for persons with heart failure within the home hospice setting	Purposive sampling. Patient participants were patients who had heart failure receiving home hospice care, family caregivers and healthcare team members 39 participants including 5 patient participants, 2 family caregivers, 24 nurses,4 social workers, 2 doctors and 2 spiritual counsellors	Qualitative descriptive study design	Semi‐structured interviews	Content analysis approach and line‐by‐line coding to create a codebook	3 themes: Trust is key to building and maintaining goals of care conversations Lack of understanding and acceptance of hospice inhibits goals of care conversations Family support and engagement promote goals of care conversations	Well‐conducted study with clearly explained methods Findings are in relation to the multidisciplinary team of which nurses were the largest group
Brogan et al. (2018)	To explore multidisciplinary healthcare professionals' perceptions and experiences of shared decision‐making (SDM) at the end of life in the home	43 participants from multidisciplinary teams from one region in the United Kingdom Purposive sample: included 12 community nurses +5 specialist nurses as well as GPs, social workers and AHPs	Qualitative design using focus groups	Focus groups with semi‐structured questions. Transcription of the discussion and theme analysis	Coding and analysis of themes. Included a blind recording by a second researcher	Participants' perceptions and experiences of SDM were related to 3 themes which were found to undermine the initiation and implementation of SDM The themes were as follows: Conceptual understanding of SDM Uncertainty in SDM processes Organizational factors which impeded the process of SDM	Well conducted study with appropriate design Took place in one region of the United Kingdom, possible limitations on generalizability
Dickson et al. (2017)	To gain insight into community nurses experiences and how they make sense of the expertise they offer in their role	Purposive sampling. Eight participants working as District Nurse Specialist Practitioners	Hermeneutic qualitative study	Semi‐structured interviews guided by a series of open‐ended questions inviting participants to tell their stories of being a DN. Participants were encouraged to keep an audio journal. 2 participants kept a journal for 6 days, 5 for 5 days and 1 did not keep a journal	Interpretative phenomenological analysis	There were 3 themes within the study. There was one theme which related closely to patient participation in decision‐making: negotiating a way in to care This theme described the way in which the DNSPQ's established a relationship of trust with patients, taking time to establish and maintain relationships. This theme described the negotiation required to reach shared decisions and allow patients and families to self‐manage The 2 other themes had some parts that were relevant to participation in decision‐making Managing complexity: relevant data from this theme was around having an understanding of the context of care and community networks to enable proactive care planning with the patient and family. Also the challenges of balancing patient autonomy and risk assessment Thinking on your feet: was around assessment and understanding the patient's need within complex and unpredictable situations	This is a well‐conducted study that has used appropriate methods This study is focused on the role of the District Nurse Specialist Practitioner and how they make sense of the expertise they offer in their role. The perspective of community nurses in relation to patient participation in decision‐making is evident in the findings within the ‘negotiating a way in to care’ theme Small number of participants and limited transferability however this is consistent with the methodology used to generate rich, thick qualitative data
Holmberg et al. (2012)	The aim of this study was to describe patients' experiences and perceptions of receiving nursing care in their private homes	Participants were recruited from 1 sub‐urban district in Sweden. Purposive recruitment via home health nurses 21 patient participants. 11 women and 10 men	Qualitative, interpretative, descriptive study	Open‐ended interviews lasting from 60 to 90 min. Participants were asked ‘what experiences do you have from receiving homecare nursing’	Data analysis was guided by Thorne's interpretative description	There were 3 themes (To be a person, To maintain self‐esteem, To have trust) but not all of these themes were relevant to participation in decision‐making 2 of the sub‐themes of To be a person were relevant which were as follows: To make choices Participating in fellowship One sub‐theme of ‘maintain self‐esteem’ was relevant which was ‘not to surrender’	Well‐conducted study that used an appropriate design
McGarry (2010)	To explore the nature of relationships between nurses and older people within the home	16 community nurses, 5 DN's, 9 RN's and 2 HCA's. Employed in the community setting between 4 months and 24 years. 13 older patients (2 male, 11 female aged from 70 to 94 years receiving care from the DN services) Purposive sampling	Ethnography Semi‐structured interviews and observation	Semi‐structured interviews, observation. This paper focuses on the interview data 29 interviews with nurse participants. 22 interviews with older people participants	Iterative approach Identification of gaps in the data, clarification in further interviews Analytic hierarchy model used for analysis and 3 stages of data management, descriptive accounts and developing explanatory accounts	2 of the 3 themes were relevant to shared decision‐making 1. Spatial boundaries: the location of care 2. The nurse patient relationship: changing boundaries of care The 3rd theme was caring boundaries: the organization of care and decision‐making was not included within this theme so will not be discussed in the review	Well‐conducted study with appropriate methods The study focused on the nature of relationships between nurses and older people in the home. Some of the findings are relevant to this review
McGarry (2008) (same research study as above)	To explore the nature of relationships between nurses and older people within the home	16 community nurses, 5 DNs, 9 RNs and 2 HCAs. Employed in the community setting between 4 months and 24 years 13 older patients (2 male, 11 female aged from 70 to 94 years receiving care from the DN services) Purposive sampling	Ethnography Semi‐structured interviews and observation	Semi‐structured interviews, observation. This paper focuses on the observational data. Researcher adopted the role of observer as partial participant 47 episodes of participant observation. Each episode was a working day of between 7 and 9 h	Iterative approach Identification of gaps in the data, and clarification in further interviews Analytic hierarchy model used for analysis and 3 stages of data management, descriptive accounts and developing explanatory accounts	Not all of the findings were relevant to participation in decision‐making. The theme ‘the meaning of ill health and illness’ was relevant as well as some of the theme ‘location of care’. The 3rd theme (the nature of relationships) was not directly relevant to this review	Well‐conducted study with appropriate methods The study focused on the nature of relationships between nurses and older people in the home. Some of the findings are relevant to this review
Millard et al. (2006)	To understand the extent to which community nurses incorporate patient involvement in decision‐making into their everyday clinical practice	22 community nurses (all female) from the DN teams. 107 patients (61 women and 46 men) Purposive sampling	Ethnography. Observation	137 interactions were observed in the home setting and clinic setting. The paper focuses only on the home setting interactions Researcher was in ‘observer as participant’ role	Examination of field diaries of observational data. Data were coded according to level and style of decision‐making undertaken by the nurse and the extent to which the patient was involved in the process of decision‐making	Variability of practice both between nurses and within the practice of individual nurses Continuum of involving/non‐involving behaviour with 5 typologies of behaviour: completely involving, partially involving, forced involving, covert non‐involving and overt non‐involving	This was a small‐scale ethnography study using appropriate methods It was based on observation and the researcher's interpretation of the observations. The methods were observation only and did not allow for the exploration of the perceptions of the participants There was a long time lag between carrying out and writing up the research
Näsström et al. (2013)	To examine how heart failure patients receiving structured home care described participation in their care	Participants were patients with heart failure receiving structured home care (as defined as a MDT with physicians and nurses who were specialists in generalist care) within the home setting 6 women and 13 men participated aged between 63 and 90 years Quota sampling used	Qualitative study	Semi‐structured interviews. The interviewer asked ‘what does it mean for you to receive homecare due to your HF?’ Then followed up with questions about participation	Interview data were analysed with an inductive approach using qualitative data analysis	5 categories describing patient participation in structured home healthcare: 1. Communication between patients and healthcare professionals 2. Accessibility to care 3. Active involvement in care 4. Trustful relations with HCPs 5. Options for decision‐making	This is a small‐scale qualitative study that has been well‐conducted with appropriate methods Small number of participants and limited transferability, however this is consistent with the methodology used to generate rich, thick qualitative data
Nilsen et al. (2021)	To expand understanding of older people living in their own homes in relation to shared decision‐making in PRN medications	Participants recruited via the head of sheltered units in 4 different areas of Norway Inclusion criteria were residents receiving help with medication who had at least one PRN medication and were able to consent to and take part in an interview 12 participants, aged between 64 and 96. 8 men and 4 women	Qualitative, explorative and interpretative design. Narrative approach	Semi‐structured interviews. Started with the question ‘if you could tell me something about your current medication use what would you tell me?’ Follow‐up questions varied depending on the response to the question	Interview data were analysed using a narrative positioning approach	Data presented as 3 narratives illustrating the way in which the participants have positioned themselves within the SDM process: 1. Passive decision maker happy to leave the decision to the nurses as long as they listen to what she says and respect her experiences 2. Active decision maker and nurses as participants. Wants to take control yet recognizes the need for help 3. Leader and owner of decisions regarding medication	This is a well‐conducted study with appropriate methods Small number of participants and limited transferability however this is consistent with the methodology used to generate rich, thick qualitative data
Olaison et al. (2021)	To explore the experiences of participation in care by frail older people with significant care needs Specific research questions were as follows: what opportunities and limitations do frail older people with significant care needs see for participation in care provided? How do the older people see opportunities and/or limitations for participation in relation to individual solutions regarding care and services provided?	The participants were a sub‐sample of a much larger intervention study in Sweden which was to investigate a focused primary care intervention and had a total number of 1600 participants, 800 in the intervention group and 800 in the control group. The sub‐sample consisted of 20 participants from the intervention group who were purposively recruited 8 women and 12 men aged between 76 and 93	Explorative with a descriptive design	Semi‐structured interviews of the patient. The spouse was present for some of the interviews and the spouses' accounts were transcribed as part of the conversation but not analysed A semi‐structured interview guide was used which focused on the participants' experiences around: everyday life/care, autonomy and own impact on healthcare/services and the future Interviews were recorded and transcribed	Content analysis was used for this study of the segments of the interviews that concerned the participants' views of analysis in their own care and treatment	There were 4 themes The theme ‘involvement on organizational levels’ was not relevant to this review The themes ‘conditions for taking part in care’, ‘involvement in direct care or treatment’ and ‘views of receiving care in relation to autonomy’ were all relevant to this review	Well‐conducted study with appropriate methods The study focused on the experiences of participation in care rather than decision‐making. Some of the findings are relevant to this review Decision‐making is not often explicitly mentioned, however, it is implicitly, for example, ‘opportunity to participate in and influence how care and treatment should be performed’ (p. 8)
Oliver et al. (2018)	To determine what elements of shared decision‐making are found in home hospice nursing visits	Secondary analysis of data from a US‐wide study where home hospice nurses were asked to audio record their visits with cancer patients. Home hospice nurses wore digital recorders during home visits for approximately 2 weeks	Secondary analysis of audio recordings of 65 home hospice visits by 65 home hospice nurses in 11 different US hospice programmes	Qualitative study using 2ndary analysis of audio recorded interviews	Analysed the data using the coding frame of Makoul's elements of shared decision‐making	All the elements of SDM were found in the interviews however they were not all used in every visit. Only 2 nurse visits combined all 9 elements. Nearly a third of visits had no elements of SDM	Well‐conducted study using appropriate methods The data are observation only and it is not possible to establish the perceptions of the nurses in relation to the elements of the shared decision‐making process The authors state that as the data relate to one visit only it does not reflect the full context and enable understanding of the already established relationship between the nurse and patient
Sundler et al. (2020) (Sweden)	The aim of this study was to explore attributes of person‐centred communication between nurses and older persons being cared for in their home	This study was part of a wider study (COMHOME study) of 188 audio recorded home visits This paper focused on 77 of the audio recordings including all visits made by RN's to patients in their homes The sample for these 77 recordings was 11 RNs and 37 older people. RNs were recruited from 4 home healthcare settings via workplace meetings (purposive sampling). The RN's recruited patient participants (snowball sampling)	Descriptive qualitative design	Data were collected by audio recording the visits. The nurses audio recorded the visits themselves from the start of the visit to leaving the home. The RNs recorded between 510 visits each lasting between 3 and 31 min	Qualitative thematic analysis was used	The 3rd finding was relevant to this literature review This theme was ‘involving in one's own healthcare’. There were 3 sub‐themes which were as follows: informing and asking for the person's view, being clear, creating a team spirit and striving for equality	This is a well‐conducted study with appropriate methods This study described attributes of person‐centred communication observed between older persons and nurses. Findings in relation to shared decision‐making are evident within the ‘involving in one's own healthcare’ theme The data are observation only and it is not possible to establish the perceptions of the nurses in relation to the elements of the shared decision‐making process
Truglio‐Londrigan (2013)	To know, understand and describe the experience of SDM in home care from the nurse's perspective	10 participants who were nurses working in home health care in the United States, who self‐identified as having had an experience of SDM	Qualitative descriptive study is described as having ‘phenomenological overtones’	2 × semi‐structured interviews where participants were encouraged to reflect on their experiences of SDM	Colaizzi's method of analysis	There were 4 themes which were as follows: Begin where the patient is Education for shared decision‐making The village and shared decision‐making Whose decision is it?	Well‐conducted study using appropriate methods Participants asked to recall their memories of their experiences which in some cases was up to 30 years ago so could have been some cognitive bias caused by memory recall Small number of participants and limited transferability however this is consistent with the methodology used to generate rich, thick qualitative data
Van Het Bolscher‐Niehuis et al. (2020)	To explore community nurses' views of self‐management, the dilemmas community nurses face when providing self‐management support for older adults and the strategies they used to solve the challenges	Purposive sampling Participants had to have been working as a community nurse for at least a year. The definition of community nurses in the study refers to nurses working within the home setting (checked the definition via email with authors) 17 female and 4 male community nurses aged between 23 and 58 years. Number of working years ranged from 1.5 to 28 years	Qualitative study	Semi‐structured interviews. Participants asked how they would describe self‐management and then the interviewer probed into the community nurses experiences by asking the nurses to describe situations where they had experienced tensions or conflict in supporting self‐management for older adults	Thematic analysis	One of the dilemmas found for nurses was ‘Striving for optimal health and well‐being’ versus ‘respecting older people's choices’ The nurses used a range of strategies to manage this dilemma which were as follows: Adapting Persuading Taking control The 2nd dilemma found for nurses was as follows: ‘stimulating self‐reliance and self‐direction’ versus ‘accepting a dependent attitude’. While very closely linked to this review it is not directly applicable as it concerns the provision of care, e.g. who will be administering insulin or phoning the doctor rather than choices around what care is provided. This is a complex theme and looking at this from a ‘participating in decision‐making perspective’ community nurses are referring to patients who have made choices around their care (whether consciously or not) that they wish to be the recipient of care delivered to them rather than to learn how to deliver the care themselves. This theme has not been included in the review	This is a well‐conducted study with appropriate methods The focus of the study is on ‘self‐management’, however, participation in decision‐making is evident within some of the findings

#### 3.3.5 Data analysis and synthesis

We carried out data analysis using reflexive thematic analysis (Braun & Clarke, 2022). One author (KM) carried out the initial analysis and development of codes/themes. Data were separated into three groups in relation to: the patients' perceptions and experiences, the nurses' perceptions and experiences and the observed experiences of the interactions between the nurse and patient. This enabled us to identify the different perspectives of the nurse and patient participants. Following familiarization, we observed patterns and relationships within the dataset and generated codes for each group. Codes were then combined for the whole dataset, and themes and sub‐themes were identified. All authors discussed and agreed on the coding.

## 4 FINDINGS

### 4.1 Characteristics of the included papers

In total, we included 14 papers for this review, which describes 13 studies with one study producing two papers (McGarry, 2008, 2010). The studies were carried out in five countries: the United Kingdom (3), the United States (4), The Netherlands (1), Sweden (4) and Norway (1). All the studies were based on a qualitative methodology including ethnography (McGarry, 2008, 2010; Millard et al., 2006), a qualitative descriptive design (Baik et al., 2020; Truglio‐Londrigan, 2013), qualitative interpretative design (Holmberg et al., 2012), generic qualitative design (Brogan et al., 2018; Näsström et al., 2013; Olaison et al., 2021; Sundler et al., 2020; Van Het Bolscher‐Niehuis et al., 2020), secondary analysis of qualitative data (Oliver et al., 2018), narrative research (Nilsen et al., 2021) and hermeneutic phenomenology (Dickson et al., 2017). A variety of methods were used to collect data including observation (Millard et al., 2006; Oliver et al., 2018; Sundler et al., 2020), semi‐structured interviews (Baik et al., 2020; Holmberg et al., 2012; Näsström et al., 2013; Nilsen et al., 2021; Olaison et al., 2021; Truglio‐Londrigan, 2013; Van Het Bolscher‐Niehuis et al., 2020), focus groups (Brogan et al., 2018), interviews, observation and document analysis (McGarry, 2008, 2010), interviews and audio diaries (Dickson et al., 2017). The sample sizes of nurse participants in the study ranged from 8 to 65 with a total of 194. The sample sizes of patient participants ranged from 5 to 107 with a total of 234. Four of the studies had small sample sizes of <20 participants (Dickson et al., 2017; Näsström et al., 2013; Nilsen et al., 2021; Olaison et al., 2021; Truglio‐Londrigan, 2013). This was appropriate for the qualitative methodologies used, enabling rich data to be obtained, however does limit the transferability of the studies. Within all the papers the researchers had addressed any potential bias of the study design and acknowledged the limitations of their studies.

The papers related to the review question in different ways. Some studies focused directly on the decision‐making process in the home, either from the community nurse or patient's perspective or observation of the decision‐making process (Brogan et al., 2018; Millard et al., 2006; Nilsen et al., 2021; Oliver et al., 2018; Truglio‐Londrigan, 2013). Other studies related to the experiences of community nurses in the home (Dickson et al., 2017) or patients’ experiences of receiving nursing care in the home (Holmberg et al., 2012; McGarry, 2008, 2010). Other studies looked at one element of shared decision‐making, for example, goal setting (Baik et al., 2020) or a closely related concept such as patient participation or self‐management (Näsström et al., 2013, Olaison et al., 2021, Sundler et al., 2020, Van Het Bolscher‐Niehuis et al., 2020).

Critical analysis of the papers' findings resulted in three themes that describe the perceptions and experiences of community nurses, and patients, towards shared decision‐making in the home. These themes were as follows: patients and community nurses value participation in decision‐making but experience a variation in practice; communication in the decision‐making process; and trust is both fundamental to and challenged by the decision‐making process. The themes are set out in Table 6 and described below.

**TABLE 6:** Themes and sub‐themes.

Theme one: patients and community nurses value participation in decision‐making in the home setting but experience a variation in practice	Sub‐theme: patients valued their right to participate in decision‐making, particularly for decisions impacting everyday lives
	Sub‐theme: nurse participants recognize the benefits of participation in decision‐making and acknowledge the challenges
	Sub‐theme: variation in the experience of shared decision‐making
Theme two: communication in the decision‐making process	Sub‐theme: being listened to and assessment of preferences and values
	Sub‐theme: information giving and assessment of understanding
	Sub‐theme: involvement of others
Theme three: trust is both fundamental to and challenged by the decision‐making process	Sub‐theme: developing trust
	Sub‐theme: tensions in the nurse–patient relationship

### 4.2 Theme one: Patients and community nurses value participation in decision‐making in the home but experience a variation in practice

Patients in these studies valued their right to be involved in decision‐making with varying preferences towards the level of engagement. Community nurses valued patient involvement in decision‐making while acknowledging the challenges. Despite these positive perceptions of shared decision‐making the observation papers found that there was a wide variation in clinical practice around decision‐making.

#### 4.2.1 Sub‐theme: Patients valued their right to participate in decision‐making, particularly for decisions impacting everyday lives

Many patient participants in these studies valued their right to participate in decision‐making (Holmberg et al., 2012; Näsström et al., 2013; Nilsen et al., 2021; Olaison et al., 2021). Some participants perceived the context of their home as enhancing their control over decision‐making (Holmberg et al., 2012; McGarry, 2010). Holmberg et al. (2012) found that engagement in decision‐making around the conditions of the home visit enhanced participants' self‐esteem. McGarry (2010) found that the participants spoke of the home as increasing their sense of autonomy to make a decision.

There was a fear expressed by participants of losing independence and control if they did not engage in decision‐making (Nilsen et al., 2021, Olaison et al., 2021). Participants in Olaison et al.'s (2021) study perceived not being able to influence the care they received as encroaching on their autonomy.

Because after all, I'm… monitoring and can speak up and have some ideas and opinions about how it shall be done. (Daniel, p. 9)

Participants had varied perceptions of their role in decision‐making and different preferences as to the extent to which they wished to be involved (Baik et al., 2020; Holmberg et al., 2012; Näsström et al., 2013; Nilsen et al., 2021). Ways in which participants felt involved in decision‐making included: preparing for home visits so they could discuss their symptoms with the nurse (Näsström et al., 2013) and making choices about when and how nursing care was delivered (Holmberg et al., 2012). Nilsen et al. (2021) identified different narratives of how patients perceived their role in decision‐making. In two of these narratives, the patient perceived themselves as taking a leading role in decision‐making: ‘the patient as owner of the decisions and leader of their care’ (p. 7) and the patient taking an active role in decision‐making, wanting to have control over the decisions while acknowledging the support needed from the nurses.

Nilsen et al. (2021) identified a third narrative of preferences towards shared decision‐making, where the patient preferred to delegate the decision‐making to the nurses. This narrative was also seen in other studies where participants seemed to prefer not to engage in decision‐making. Some participants in Näsström et al.'s (2013) study chose to delegate responsibilities for decisions to healthcare professionals. In McGarry's (2008) study, one participant described herself as doing as she is told:

I will do whatever you tell me, you are the nurse. (Mrs H, p. 88)

However, while the preference may be to delegate the decision‐making process, there is some evidence within the studies that if a decision matters to the person and impacts on their daily life, it is important for them to speak up (McGarry, 2008, 2010; Näsström et al., 2013; Nilsen et al., 2021). McGarry (2008) suggested that patients made choices when to discuss or challenge their treatment with the community nurses, particularly when it affected their everyday lives. Patients may experience specific barriers that make it difficult for them to have more active participation in decision‐making, such as a perception of not wanting to be a burden (Nilsen et al., 2021), being ‘*too old*’ (Person with heart failure 2, Baik et al., 2020, p. 930) or cultural barriers (Baik et al., 2020; Nilsen et al., 2021).

#### 4.2.2 Sub‐theme: Nurse participants recognize the benefits of participation in decision‐making and acknowledge the challenges

Four papers included findings relating to the perceptions of nurse participants towards patient participation in decision‐making (Brogan et al., 2018; Sundler et al., 2020; Truglio‐Londrigan, 2013; Van Het Bolscher‐Niehuis et al., 2020). Some nurses valued shared decision‐making (Truglio‐Londrigan, 2013; Van Het Bolscher‐Niehuis et al., 2020) acknowledging that patients want to be involved in decisions about their care and recognized the benefits of shared decision‐making. For example, one participant in Truglio‐Londrigan's (2013) study described the positive benefits of shared decision‐making to the patient in her care as:

how much more happy she became and how much more confident she seemed to be. (Rebecca, p. 2890)

Some participants acknowledged their role in empowering patients (Sundler et al., 2020) and supporting patients in ‘*finding their voice*’ to actively engage in decisions (Truglio‐Londrigan, 2013, p. 2891). Participants in Van Het Bolscher‐Niehuis et al.'s (2020) study spoke of ‘giving them a little more confidence’ (participant R15, p. 200) to engage in their care more proactively.

Other participants acknowledged the tensions surrounding shared decision‐making. The participants in Brogan et al.'s (2018) study described the challenges of working within a community environment that was task‐orientated and time‐pressured and felt that shared decision‐making is ‘a desirable but luxurious therapeutic conversation’, (p. 126). The concern about time is partially reflected in Oliver et al.'s (2018) observational study. The two visits where the nurses used all the elements of Makoul and Clayman's (2006) framework of shared decision‐making, were longer than average. However, there were other visits that were longer, which did not use all the elements.

#### 4.2.3 Sub‐theme: Variation in experience of shared‐decision making

The research papers where the decision‐making interaction had been observed found a variety of practices suggesting different values and preferences towards shared decision‐making by community nurses. Millard et al. (2006) identified five different categories of behaviour of community nurses on a continuum ranging from complete involvement through to non‐involvement of patients. Only two of the 22 community nurse participants were consistent in practice and completely involved patients in decision‐making. Oliver et al.'s (2018) also found wide variation in practice between nurses. Only two (3%) of nurse visits contained all nine elements of Makoul and Clayman's (2006) framework of shared decision‐making, and another five nurse visits (8%) demonstrated all but one element. However nearly a third (31%) of the nurse visits contained no elements of shared decision‐making.

### 4.3 Theme two: Communication in the decision‐making process

Communication in the decision‐making process was perceived as beneficial to nurses and patients to facilitate sharing of decisions. Patients wanted to be listened to and nurses acknowledged the importance of hearing the patient's values and preferences as part of the assessment process. The provision of information was valued by both patients and nurses. Nurses acknowledged the need to involve others in the decision‐making process.

#### 4.3.1 Sub‐theme: Being listened to and assessment of preferences and values

Being listened to was important to patient‐participants. They wanted to be respected as a unique individual and for the nurse to listen and be interested in them (Holmberg et al., 2012; Näsström et al., 2013). This included a holistic rather than fragmented approach and a two‐way dialogue with the nurse (Näsström et al., 2013; Nilsen et al., 2021; Olaison et al., 2021; Sundler et al., 2020).

that they talked to me. That they asked me what I want and do not want…. (participant P4, Näsström et al., 2013, p. 1390)

Patient participants perceived the home setting as enabling space for conversations, particularly if the nurse had sufficient time and a holistic rather than task‐orientated approach (Näsström et al., 2013).

Nurse participants valued getting to know patients and listening to their stories (Brogan et al., 2018; Dickson et al., 2017; McGarry, 2008, 2010; Truglio‐Londrigan, 2013). Nurse participants in Brogan et al.'s (2018) study found that working in the home facilitated their understanding of the patient. Similarly, participants in McGarry's (2008) and McGarry's (2010) studies articulated their values around the home setting, enabling an increased understanding of older people and their life history.

…she's been able to tell me all about her past life…what things were like then… (participant RN4, p. 86, McGarry, 2008)

For the nurse participants, getting to know the patient was part of the assessment process (Dickson et al., 2017; McGarry, 2008, 2010; Truglio‐Londrigan, 2013) and this knowledge contributed towards decision‐making. A participant in Dickson et al.'s (2017) study described the process of finding out the patient and carer's understanding of their situation as ‘doing the dance around’ (Steph, p. e457) illustrating the work that she did to understand the person's situation and to come to a shared decision.

In Baik et al.'s (2020) study, there were sometimes challenges for nurse participants in gaining an understanding of what matters to patients when the nurse and patient did not speak the same language or had different cultural understandings.

That (language discordance) has been difficult for me. We had to have an interpreter and it's not really comforting for the patient. (Nurse 1, p. 929)

Oliver et al. (2018) observed the most frequent type of communication relating to the decision‐making process was defining the problem or assessment. There were 271 types of problems identified by the nurse participants, approximately four per visit, relating to physical, psychosocial and financial issues, for example, pain, fatigue or anxiety.

Sundler et al. (2020) found that nurse participants asked open questions such as ‘What do you think’ (p. 7) to elicit patients' preferences. Oliver et al. (2018) found that patient preferences and values were heard in 60% of the nursing encounters. The preferences related to treatment, for example, which medication to take; patient values, for example, how much pain could be tolerated; and logistics, for example, how to obtain medication.

#### 4.3.2 Sub‐theme: Information giving and assessment of understanding

The nurse participants perceived education and information giving as important in supporting patients to engage in the decision‐making process (Baik et al., 2020; Brogan et al., 2018; Truglio‐Londrigan, 2013). Sundler et al. (2020) found that nurses informed patients about the care they had planned and any actions required. In Baik et al.'s (2020) study, information giving included providing information about the home hospice service and clarifying expectations. Oliver et al. (2018) found that information‐giving was one of the most used elements of shared decision‐making, with over two‐thirds of nurses giving information and recommendations. Information most commonly related to symptom management and changes in medication.

Sometimes there were challenges with information giving, for example, delayed or fragmented information exchange (Brogan et al., 2018). In Oliver et al.'s (2018) study under half of the nurses presented information about specific options in relation to palliative care symptoms. Nurses were inconsistent concerning the discussion of risks and benefits, with these being discussed in under a tenth of home visits.

The participants in Truglio‐Londrigan's (2013) study perceived education as an ongoing process.

I would say to them, you know the last time we discussed this you said you would try such and such. Did you? (Rebecca, p. 2889)

It was also important to participants in Truglio‐Londrigan's (2013) study that they checked patient understanding of the information. Examples of ways they facilitated patient understanding included taking lab reports to the home to explain to patients and using a mirror to show a patient their wound. There were challenges with checking understanding, particularly when the patient was unwell or there were language or cultural barriers (Baik et al., 2020; Brogan et al., 2018; Truglio‐Londrigan, 2013; Van Het Bolscher‐Niehuis et al., 2020). Oliver et al. (2018) found that the assessment of patient understanding was the least used element of shared decision‐making in their study, leading to questions as to the effectiveness and consistency of information‐giving in developing patient understanding.

Interestingly very few patient participants discussed the importance of information. One participant in Näsström et al. (2013) study felt that information was very important in helping him to manage his condition.

First give information to the patient, that is the alpha and omega… then you know what you have and what you are dealing with. (participant P7, p. 1388)

Some participants felt insecure in relation to their own knowledge which could be a barrier to the nurse–patient relationship (Näsström et al., 2013; Olaison et al., 2021).

And I don't think I've ever come across that they say “no, no it's not like that”, they take it in, or maybe they are so smart that they keep a straight face and let me carry on with my ideas. (Stephan, Olaison et al., 2021, p. 7)

Some participants discussed the ways they researched for information about their condition. One participant in Nilsen et al.'s (2021) study discussed how she obtained her information from the internet. Some participants in Näsström et al.'s (2013) study read about their treatment and side effects.

#### 4.3.3 Sub‐theme: Involvement of others

Community nurses acknowledged that the decision‐making process involved others, including family members and the multi‐professional team (Brogan et al., 2018, Truglio‐Londrigan, 2013, Van Het Bolscher‐Niehuis et al., 2020). The patient participants did not discuss in any of the studies whether it was important to them to involve their family members or the multi‐disciplinary team.

The nurse participants felt that it was important to involve the family members in the decision‐making process (Brogan et al., 2018, Truglio‐Londrigan, 2013). Some nurses would follow the patient's lead and would only involve the family if the patient did (Brogan et al., 2018 p. 126).

Some community nurses acknowledged the importance of the multidisciplinary team in the decision‐making process (Brogan et al., 2018; Truglio‐Londrigan, 2013; Van Het Bolscher‐Niehuis et al., 2020). Involvement of the multidisciplinary team gave confidence to the nurses in the decisions made, enabled different perspectives, and was perceived as ensuring accountability of the decisions made.

There were several barriers that inhibited the involvement of the multi‐professional teams, including a lack of continuity of staff, large and geographically spread out teams and a lack of clarity over responsibilities for decision‐making (Brogan et al., 2018).

### 4.4 Theme three: Trust is both fundamental to and challenged by the decision‐making process

Developing a trusting relationship was perceived by both patient and nurse participants as fundamental to the decision‐making process. However different perspectives about the decision could lead to tensions occurring in the nurse–patient relationship challenging the shared nature of decision‐making.

#### 4.4.1 Sub‐theme: Developing trust

Patient participants felt that trusting and having confidence in the nurse enabled their participation in the decision‐making process (Baik et al., 2020; Näsström et al., 2013; Olaison et al., 2021).

Nurse participants also valued the nurse–patient relationship as supporting patients to participate in decision‐making (Baik et al., 2020; Brogan et al., 2018; Dickson et al., 2017; Truglio‐Londrigan, 2013). Participants articulated how they slowly developed a trusting relationship through negotiation (Dickson et al., 2017; Truglio‐Londrigan, 2013). Through negotiation, the nurses were able to come to an agreement which might be to gain access to the person's home or to compromise over a treatment decision.

I basically tapped on her door and told her who I was… I said I would respect her boundaries and she slowly let me into the home. (Zanzibar, Truglio‐Londrigan, 2013, p. 2888)

The continuity of patients' relationships with nurses was perceived as necessary for developing trust and enabling participation (Näsström et al., 2013, Olaison et al., 2021). Olaison et al. (2021) found that the continuity of healthcare relationships seemed to enhance the person's ability to have control over their care.

Conversely, when a patient did not trust the nurse, it was more difficult for the patient to participate and share information. If the patient does not trust the nurse they may refuse to let them into the home or ask them to leave as described by a participant in Holmberg et al. (2012):

The nurse rushed in and there I stood, I couldn't turn around or anything… Then he said “I'm in a hurry.” Well if you are in a hurry you shouldn't come here, I said. (participant not named, p. 707)

Näsström et al. (2013) found that where there was a lack of trust in the shared decision‐making process, patients would not ask questions and might not describe their symptoms fully. Similarly, Brogan et al. (2018), found that if the trusting relationship was not in place, it was difficult for nurses to discuss important issues with the patient and to encourage participation.

Power dynamics influenced the trusting relationship between the nurse and the patient. Sundler et al. (2020) observed that where the nurses seemed aware of the potential power imbalance and communicated to reduce power imbalances, a more equal partnership developed, enabling more sharing of decisions. Similarly, Millard et al. (2006) found that when community nurses demonstrated interest in the person, it enabled the nurse and patient to engage more equally in the partnership. This facilitated the dialogue between the nurse and patient, empowering the patient to be actively involved in making decisions.

#### 4.4.2 Sub‐theme: Tensions in the nurse–patient relationship

There was evidence of tensions that could occur when there were different perspectives on the decision to be made (Millard et al., 2006; Truglio‐Londrigan, 2013; Van Het Bolscher‐Niehuis et al., 2020). In Van Het Bolscher‐Niehuis et al. (2020) and Truglio‐Londrigan's (2013) studies, nurse participants articulated feelings of discomfort, frustration or internal conflict around patients making choices that they disagreed with. Van Het Bolscher‐Niehuis et al. (2020) identified two dilemmas for nurses concerning the decision‐making process around patient self‐management. First, the nurses wanted to promote good health and well‐being of older adults, yet wanted to respect older adults' preferences. Second, nurses felt frustrated when they experienced patients making the decision that they would prefer the nurse to provide the care, rather than learn to be independent, for example, the administration of insulin.

Sometimes, where there was a difference of opinion, the nurse would take control of the decision‐making (Millard et al., 2006; Van Het Bolscher‐Niehuis et al., 2020). Millard et al. (2006) observed a nurse refusing to change the wound management treatment in response to the patient's preference (p. 147). Nurse participants in Van Het Bolscher‐Niehuis et al.'s (2020) study discussed their perspective on how they sometimes took over the decision‐making if they felt the alternative was unsafe, prioritizing beneficence over the patient's right to make a decision.

If it is no longer sensible to let them decide or do it themselves because they form an immediate danger to themselves or their environment then I intervened…Then in any case I did not fail in my duty. (Participant R13, p. 200)

Sometimes the nurse would try to persuade a patient towards a decision with the support of family members or other health professionals (Van Het Bolscher‐Niehuis et al., 2020). Other times, nurses would try to persuade patients with a focus on the negative consequences of the decision, (Van Het Bolscher‐Niehuis et al., 2020).

Then I say: if you break a hip you will have to go to the hospital and afterwards you will probably no longer be able to live independently. (Participant R19, p. 200)

Millard et al. (2006) found that sometimes the nurse would not initially involve the patient, but when the patient asserted themself and challenged the nurse's decision the nurse would be forced to involve the patient.

When challenged twice by the patient she offered alternatives, one of which the person chose which suited her lifestyle better. (p. 146)

Sometimes when the patient makes a decision that the nurse does not agree with, the nurse goes through a process of accepting and coming to terms with the decision that was made (Truglio‐Londrigan, 2013; Van Het Bolscher‐Niehuis et al., 2020). This included questioning their own decision and discussing with the wider multi‐professional team for support and to check accountability (Truglio‐Londrigan, 2013; Van Het Bolscher‐Niehuis et al., 2020).

We need to validate whether we were making the right decision, whether the approach was correct… so it took a bit of soul searching. (Marlou, Truglio‐Londrigan, 2013, p. 2890)

## 5 DISCUSSION

This review explored the perceptions and experiences of community nurses and patients towards shared decision‐making in the home, illustrating that involvement in decision‐making is important to both patients and nurses. The key findings were that shared decision‐making is valued by patients and community nurses and that communication and trust in the relationship are perceived as critical. However, shared decision‐making does not always happen in practice and nurses sometimes report overriding the patient's decision; this affects the trust in the relationship.

Shared decision‐making is a goal to which nurses should aspire. We have presented shared decision‐making as both evolving from and an extension of, informed consent, acknowledging the developing legal, ethical and professional requirements of the consent process which is recognized in many countries. Consent can be applied within a shared decision‐making framework. In the United Kingdom, the Montgomery judgement (Montgomery v. Lanarkshire Health Board, 2015) put shared decision‐making at the centre of practice. The papers included within the review span two decades (from 2001 to 2023), in which time the process of shared decision‐making has become more established. However, reference to shared decision‐making has not varied between the studies. There was a limited acknowledgement by the participants of the legal, professional or ethical basis of shared decision‐making. Patient participants in these studies expressed valuing their autonomy to speak up and participate in decision‐making, though reporting varying preferences as to the extent of involvement. Some nurses seemed proactive in supporting the patient to develop their voice, and to speak up. In contrast, others seemed to value the ‘right’ decision, in their professional opinion, being made, prioritizing beneficence over the patient's right to make their own choices. Where the nurse recognized and enabled the patient's right to make a decision, this could lead to challenges for the nurse concerning moral distress experienced when what they perceived was right for the patient clashed with the patient's preferences. Equally, insisting on a course of action without agreement from the patient could affect trust. This suggests that shared decision‐making is complex. It has long been recognized that nurses find it challenging when patients refuse treatment that is in their best interests (Aveyard, 2005; Aveyard et al., 2022). Some nurses were unsure about their accountability in this situation, needing reassurance from other healthcare professionals.

Our finding that participants' perception of communication being central to shared decision‐making echoes Elwyn et al.'s (2017) ‘3 talk model’ of shared decision‐making, a valuable framework to consider the current findings. In this model, shared decision‐making is described as a cycle that includes ‘team talk’, ‘options talk’, ‘decision talk’, ‘active listening and deliberation’. We found that ‘team talk’ is the predominant focus of most conversations but does not lead to options talk and decision talk which might be the anticipated outcome. We suggest that while community nurses focus on relationship building and overall patient care, this falls short of articulating options and decisions. The perceptions of both patients and nurses are that they value the patient–nurse relationship, including the two‐way dialogue and development of trust. ‘Active listening’ is discussed by patient participants as important to them within the current findings. While there was some discussion of ‘options talk’ within the papers, this was limited. Theoretically, information giving was valued by nurses, however, in practice, there was variability in the extent to which information about the risks and benefits of treatment options was delivered and patients' understanding assessed (Oliver et al., 2018). Similarly, Aveyard et al. (2022) found that while information giving was perceived as an accepted part of nursing care (team talk), this was focused on informing the patient about their care rather than providing information to facilitate decision‐making. Access to information is considered a social determinant of health which impacts on health outcomes (Graham et al., 2024). With the development of social media and the proliferation of information online of variable quality, the nurse not only needs to provide good quality information within the shared decision‐making process but also to support people in understanding and applying health information to their own situation (Nutbeam & Lloyd, 2021). Although the importance of information giving is well established, we have concluded that nurses’ and patients’ talk falls short of the detailed discussion of options and subsequent decisions, which might be expected if patient participation in decision‐making was truly undertaken.

We found little clarity within the papers about how the decision is made and/ or by whom and how the actual interaction between the nurse and patient plays out to enable a decision to be made. Similarly, Marriott‐Statham et al.'s (2023) review of shared decision‐making with older people found a gap concerning the actual practicalities of shared decision‐making and how the decision was made. Furthermore, while it could be assumed (and indeed anecdotally is often stated) that the home environment facilitates decision‐making, we did not find evidence of this. Some nurses did indeed find that they gained an increased understanding of the patient, and some patients found that they could speak up more in the home, but this was not conclusive and more research is needed to develop our understanding of this area.

### 5.1 Limitations of the review

There were several limitations to this review. The review has a broad focus on decision‐making rather than specifying a particular type of decision. The rationale for this was that there are limited studies carried out in the home setting and the need to capture all the research relevant to the review question. We acknowledge that taking this broad approach may have meant that there are nuances in relation to specific treatment decisions that were missed. However, while the type of decision might be different, the process of decision‐making is often similar as was found within these studies.

The 13 studies that met the inclusion criteria were conducted across five countries, nine studies were carried out in Europe and four in the United States. These studies therefore do not represent the complete global picture relating to the perspectives and experiences of patients and nurses of participation in decision‐making in the home setting. Interaction during the decision‐making process is a culturally specific process, and differences between the perspectives of nurses and patients within different countries, and from different backgrounds, may not have been fully captured within this study.

The studies were small, and whilst some related directly to the review question, others were focused on broader topics and only partial findings were relevant to this review. Various terms were used to describe participation in decision‐making, and there are subtle differences between the terms used, creating complexity in determining which studies to include. The differing aims and methodologies of the studies made generalizability across the studies challenging. Despite this, the integrative review methodology together with reflexive thematic analysis allows for a range of studies with different methodologies to be combined.

## 6 CONCLUSIONS

In this review, we have highlighted that while patients and nurses value shared decision‐making, the extent to which this takes place in practice is variable. We found little evidence that the process is fully embedded into nursing practice within the home setting. A trusting nurse–patient relationship is perceived as paramount to enabling shared decision‐making by the participants of these studies. Yet this relationship may be challenged by different opinions about the situation, or treatment options. The findings suggest that nurses in these studies seemed to value beneficence over autonomy. We have highlighted a gap in the findings as to the process of how decisions are made, by whom and how the actual interaction between the nurse and patient plays out within the home to enable a decision to be made. Further research is required to understand what happens within the decision‐making process. Gaining more insight into how the interaction between the individual in the home and the community nurse contributes to patient engagement in decision‐making will promote shared decision‐making and in turn improve patient care.

## AUTHOR CONTRIBUTIONS

All authors have agreed on the final version and meet at least one of the following criteria (recommended by the ICMJE*): (1) substantial contributions to conception and design, acquisition of data, or analysis and interpretation of data; (2) drafting the article or revising it critically for important intellectual content. Made substantial contributions to conception and design, or acquisition of data, or analysis and interpretation of data: KM, LM, MW and HA. Involved in drafting the manuscript or revising it critically for important intellectual content: KM, LM, MW and HA. Given final approval of the version to be published. Each author should have participated sufficiently in the work to take public responsibility for appropriate portions of the content: KM, LM, MW and HA. Agreed to be accountable for all aspects of the work in ensuring that questions related to the accuracy or integrity of any part of the work are appropriately investigated and resolved: KM, LM, MW and HA.

## FUNDING INFORMATION

This research received no specific grant from any funding agency in the public, commercial or not‐for‐profit sectors.

## CONFLICT OF INTEREST STATEMENT

No conflict of interest has been declared by the authors.

### PEER REVIEW

The peer review history for this article is available at https://www.webofscience.com/api/gateway/wos/peer‐review/10.1111/jan.16345.

## Supporting information


Data S1.

## Data Availability

Data sharing not applicable to this article as no datasets were generated or analysed during the current study.
